# Pyoderma Gangrenosum as the First Manifestation of Inflammatory Bowel Disease: A Case Report

**DOI:** 10.1155/crgm/2028354

**Published:** 2026-01-13

**Authors:** Nam Hoai Nguyen, Tan Thi Tran, Trang Thu Khuc, Yen Thi Lo, Ha Thi-Ngoc Doan, Hieu Van Nguyen, Long Cong Nguyen

**Affiliations:** ^1^ Gastroenterology and Hepatology Center, Bach Mai Hospital, Hanoi, Vietnam, bachmai.gov.vn; ^2^ University of Medicine and Pharmacy, Vietnam National University, Hanoi, Vietnam, vnu.edu.vn

**Keywords:** case report, inflammatory bowel disease, pyoderma gangrenosum

## Abstract

Approximately 50% of patients with inflammatory bowel disease (IBD) exhibit extraintestinal manifestations, among which skin and mucosal lesions are common. However, pyoderma gangrenosum occurs in only 1%‐2% of IBD patients. We report the case of a 23‐year‐old male patient who was admitted to the hospital with pyoderma gangrenosum that was unresponsive to conventional treatments. The patient did not exhibit any gastrointestinal symptoms. We conducted diagnostic investigations, excluding causes such as infections, vascular occlusions, and systemic diseases. The patient did not respond to systemic antibiotic therapy and local care. With mild thickening of the colon wall observed on a CT scan, we decided to perform a colonoscopy, which revealed lesions suggestive of chronic ulcerative colitis. Histopathological examination of the colon tissue confirmed the diagnosis. With the diagnosis of early onset chronic ulcerative colitis presenting with extraintestinal manifestations, we decided to treat the patient with the biological drug infliximab. After 3 months of treatment, the pyoderma gangrenosum lesions healed. In summary, this clinical case indicates that pyoderma gangrenosum can be an initial and rare manifestation of IBD.

## 1. Introduction

Extraintestinal manifestations are also common in patients with inflammatory bowel disease (IBD), with frequencies ranging from 6% to 47%, depending on the study [1]. These symptoms are more prevalent in younger patients and are more common in Crohn’s disease than in ulcerative colitis. Approximately 25% of patients experience extraintestinal symptoms an average of 5 months before being diagnosed with IBD. Over 30 years of follow‐up after IBD diagnosis, about 50% of the patients have at least one extraintestinal symptom. Commonly affected organs include joints, skin, and eyes, while less common manifestations include liver (primary sclerosing cholangitis), thrombotic events, or renal involvement [2]. In one study, about 63% of the patients had one extraintestinal symptom, 26% had two, 5% had three, 2% had four, and 3% had five extraintestinal symptoms [1]. We herein report a case of pyoderma gangrenosum in a patient with IBD.

## 2. Case Presentation

A 23‐year‐old male patient was admitted to the hospital due to ulcerative skin lesions on the lower leg. The patient had a history of a perianal abscess 1 year prior, which had been treated with incision and drainage, followed by recovery. Three months before admission, the patient developed pustules on the outer lower third of the left leg and self‐medicated with an unspecified topical treatment, which resolved the issue. However, 1 month later, the pustules recurred at the same site but spread more extensively, forming an abscess, and new lesions appeared on the upper third of the sternum and under the chin. The patient sought treatment at a provincial hospital, where he received antibiotics, incision, and drainage of the lesions, with successful skin grafting of the defects, and he was subsequently discharged. Nevertheless, the lesions recurred more severely, with increased pus discharge and a fever of 38.5°C, prompting admission to the Center for Tropical Diseases.

**Figure 1:**
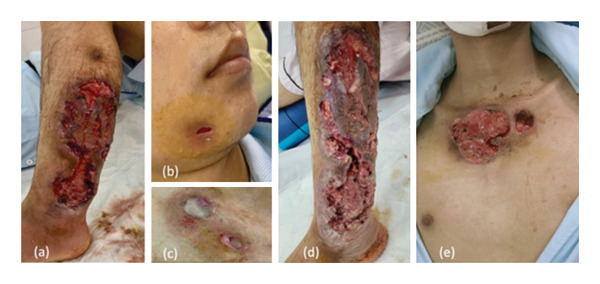
Left image: skin lesions upon hospital admission on the anterior aspect of the left shin (a), lateral aspect of the left jawbone (b), and one‐third of the clavicle (c). In the right image, lesions worsen after 4 days, showing the anterior aspect of the left shin (d) and one‐third of the clavicle (e).

Upon admission, the patient was alert but febrile and exhibited signs of infection, having lost 10 kg since the onset of the disease. The skin lesion on the outer left lower leg measured 10 × 20 cm, was deeply ulcerated, had extensive tissue loss, and was discharging copious amounts of pus with necrotic wound edges. Additionally, skin lesions on the upper third of the sternum measuring 3 × 3 cm and on the outer left mandible measuring 1 × 0.5 cm had similar characteristics (Figure 1). The patient had no other significant. He had one bowel movement per day with slightly loose stools and no mucus or blood.

Blood tests revealed a significant infection, markedly elevated peripheral white blood cell count (25.2 10^9^/L, 83.9% neutrophils), and increased CRP at 213.6 (Table 1).

**Table 1:** Laboratory test results.

Index	Result	Normal range
RBC (T/L)	4.02	4.5–5.9
Hemoglobin (g/L)	**92**	135–175
Hematocrit	0.294	0.41–0.53
WBC (G/L)	**25.2**	4.0–10.0
NEU (%)	**83.9**	45–75
PLT (G/L)	462	150–400
Urea (mmol/L)	4.9	3.2–7.4
Creatinine (μmol/L)	78	59–104
Glucose (mmol/L)	7.6	4.0–6.0
HbA1c (%)	5.2	4.0–6.0
Protein (g/L)	79	66–87
Albumin (g/L)	38.2	35–52
Total bilirubin (mcmol/L)	1.8	< 11.7
LDH (U/L)	167	240–480
ALP (U/L)	84	40–129
Serum ion (mcmol/L)	**3.7**	8.1–28.6
Ferritin (ng/mL)	159.4	30–400
CRP (mg/dL)	**213.6**	< 5
ESR 1/2 h (mm)	15/38	0–10/0–20
IgA (mg/dL)	220	70–400
IgG (mg/dL)	**1959**	700–1600
IgM (mg/dL)	178	40–230
Interleukin‐6 (pg/mL)	**41.04**	< 7
CD3^+^ T cells (cells/mL)	440	800–2300
CD4^+^ T cells (cells/mL)	230	400–1300
CD8^+^ T cells (cells/mL)	135	250–800

*Note:* Bold values represent values that are higher or lower than normal values.

The urinalysis (random sample) showed no leukocyturia, negative nitrites, and a proteinuria of 0.15 g/L. Viral tests were negative for HBsAg, anti‐HCV, and anti‐HIV. Autoimmune antibody tests revealed a positive anti‐dsDNA 1 (+) and a suspected lupus anticoagulant. Most other antibodies were negative, including antinuclear antibody (ANA), anticardiolipin, anti‐Sm, pANCA, cANCA, anti‐beta2‐glycoprotein, anti‐SS‐A, and anti‐SS‐B. Other test results included a positive direct Coombs test 1 (+) and a negative indirect Coombs test.

Diagnostic imaging results were as follows: Doppler ultrasound of the lower extremities showed no arterial stenosis or venous thrombosis. Cardiac and abdominal ultrasounds were regular. Peripheral lymph node ultrasound revealed lymph nodes at the bilateral mandibular angles and inguinal regions with preserved hilum structure. Chest x‐ray and computed tomography (CT) of the chest showed no abnormalities.

Regarding the leg lesion, an x‐ray of the shin showed no abnormalities in the bones or joints. For the chest wall lesion, MRI indicated inflammation of the bilateral first costosternal joints, with edematous soft tissue and subcutaneous tissue inflammation around the sternoclavicular and first costosternal joints. Abdominal CT scan revealed mild thickening (5 mm) of the descending and sigmoid colon walls and multiple mesenteric lymph nodes, the largest measuring 10 × 9 mm.

**Figure 2:**
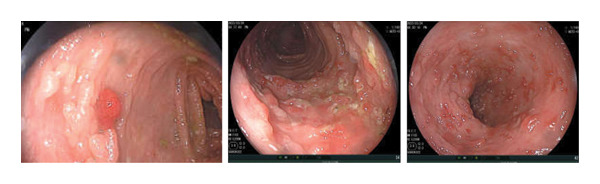
Endoscopic images of the ascending colon (left), transverse colon (middle), and sigmoid colon (right).

The patient was diagnosed with sepsis, cellulitis, and multiple soft tissue abscesses. Treatment included a combination of antibiotics: imipenem/cilastatin 3 g/day, vancomycin 2 g/day, levofloxacin 750 mg/day intravenously, and cotrimoxazole 400/80 mg, nine tablets/day orally. Additionally, the lesions were cleaned daily; however, the skin lesions continued to worsen and spread (Figure 2). Biopsies of the skin lesions on the leg, face, and chest revealed epidermal keratinocyte necrosis, with numerous neutrophils, histiocytes, and macrophages infiltrating the dermis, forming microabscesses with scattered multinucleated giant cells. No vasculitis or specific lesions were observed. Histology suggested acute purulent inflammation but could not rule out pyoderma gangrenosum.

Microbiological cultures of sputum, blood, and pus from all three skin lesion sites did not detect any bacteria, fungi, or tuberculosis pathogens.

Despite the absence of symptoms such as bloody diarrhea, due to the presence of necrotizing skin inflammation and mild thickening of the colon wall observed on the CT scan, the patient was indicated for a colonoscopy. The endoscopic findings showed normal ileocecal valve and terminal ileum. The ascending colon had multiple pseudopolyps. The transverse colon exhibited superficial ulcers interspersed with areas of healed mucosa and pseudopolyps. The descending colon through to the sigmoid colon displayed widespread mucosal inflammation, friability, absent vascular pattern, and numerous pseudopolyps. The rectum showed mild erythema, and there were no fistulas or abscesses in the anal canal.

**Figure 3:**
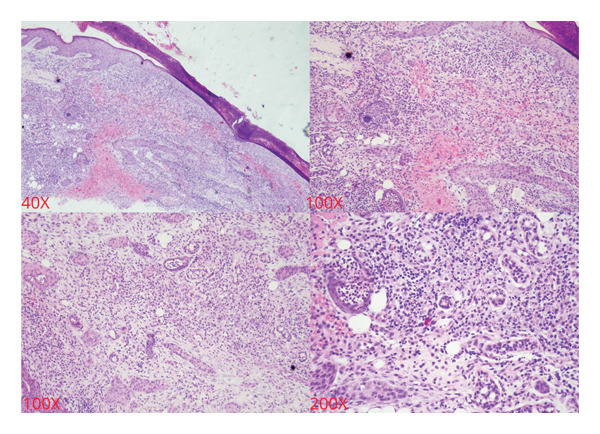
Biopsy of tissue samples from the transverse and sigmoid colon revealed epithelial degeneration and desquamation.

Biopsy of tissue samples from the transverse and sigmoid colon revealed epithelial degeneration and desquamation (Figure 3). Several glands showed changes, becoming twisted, atrophied, or dilated, with a loss of parallel arrangement. The stroma was infiltrated with numerous lymphocytes, plasma cells, and scattered eosinophils. These histological findings suggest chronic ulcerative colitis.

**Figure 4:**
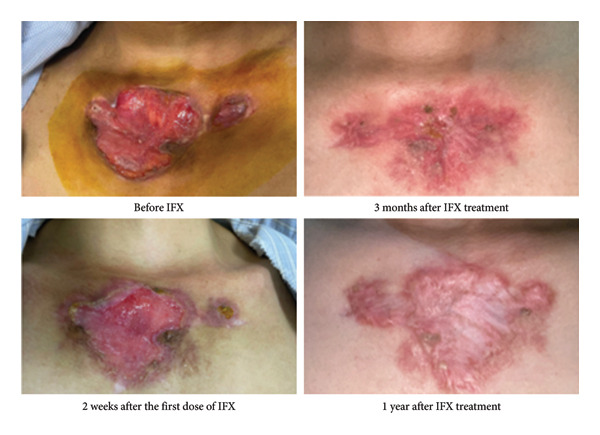
Skin lesions on the chest, gradually regressing over time after treatment with infliximab.

**Figure 5:**
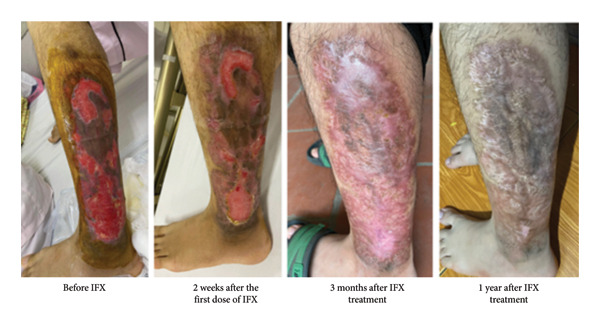
Progressive healing of left lower leg ulcers following infliximab treatment.

Based on the results from laboratory tests, endoscopic examinations, imaging, and histological analysis, we diagnosed the patient with ulcerative colitis presenting with pyoderma gangrenosum. Given that IBD with extraintestinal manifestations is classified as severe, the patient was immediately prescribed the TNF‐α inhibitor infliximab according to the standard regimen: 5 mg/kg body weight at weeks 0, 2, and 6, followed by every 8 weeks. The skin lesions were treated with saline washes and local anti‐infective measures, gradually improving over time (Figures 4 and 5).

## 3. Discussion

Regarding mucocutaneous manifestations in IBD patients, the current literature divides them into five mechanistic groups, including (1) specific lesions with histological characteristics similar to IBD, (2) reactive skin lesions, (3) lesions related to IBD, (4) secondary skin lesions due to IBD treatment, and (5) mucocutaneous lesions due to IBD‐related malnutrition consequences [3, 4].

Skin lesions with histological characteristics similar to IBD are the result of widespread inflammation extending from the intestinal tract to the perianal area, such as perianal fissures (20%–25% of Crohn’s patients [5]), anal ulceration (2%–5% [3]), perianal fistulas (6%–34% [5]) or perianal skin tags. Alternatively, lesions may spread to the oral cavity, causing ulcerations (8%‐9% [5]). Histologically, these lesions exhibit transmural and granulomatous inflammation, similar to Crohn’s disease. Some cases may present with hemorrhagic patches, ulcers, or mass‐like lesions extending beyond the gastrointestinal tract, often in the lower limbs or intertriginous areas such as the axilla, groin, or genitalia, with histological features of IBD [5].

Reactive skin lesions in IBD share similar pathogenic mechanisms but do not exhibit histological characteristics of IBD. These include pyoderma gangrenosum, oral mucosal pyoderma, Sweet’s syndrome, bowel‐associated dermatosis arthritis syndrome (BADAS), aphthous ulcers, and sterile abscess syndrome [5]. The characteristic pathogenic mechanism involves sterile neutrophilic inflammation in target tissues, characterized by overproduction of IL‐1b, leading to release of proinflammatory cytokines through neutrophils, also known as neutrophilic dermatosis. Pyoderma gangrenosum may occur in 1%‐2% of IBD patients [3]. In contrast, 50% of the patients with pyoderma gangrenosum have IBD [3]. It is more common in ulcerative colitis patients (5%–20%) than in Crohn’s disease patients, is more prevalent in females, and often precedes IBD onset [3]. Initially presenting as a sterile pustule or abscess, it quickly ruptures into painful ulcerations with well‐defined borders, progressing in size or regressing spontaneously, leaving scars. Pyoderma gangrenosum lesions may surround the artificial anus. Differential diagnosis should exclude other conditions such as infectious dermatitis, vasculitis, or malnutrition‐related ulcers [3]. Another rare but closely related IBD‐associated condition (70% of the patients) is pyodermatitis‐pyostomatitis vegetans, presenting as edematous, inflamed oral mucosa with multiple pustules prone to rupture, causing hemorrhagic ulcers [5]. Tongue and palate involvement is less common. Mucosal lesions may also occur in other mucosal surfaces such as the vagina, nose, and rarely in the eyes. Histologically, there is acanthosis with superficial ulceration and neutrophilic infiltration within or beneath the epithelium. Sweet’s syndrome manifests with fever, peripheral neutrophilia, and erythematous plaques or nodules, 20% showing annular configurations, with histological evidence of neutrophilic infiltration spreading [3, 5]. Lesions are more common on the face and extremities than on the trunk. BADAS presents with fever, pseudoflu symptoms, myalgia, nondeforming polyarthritis, and polymorphic skin lesions resembling pyoderma gangrenosum or Sweet’s syndrome [5]. Aphthous ulcers in the oralpharyngeal region may occur in 10% of IBD patients, with histological features of neutrophilic infiltration at mucosal surfaces like the gums, lips, buccal mucosa, tongue, and oropharynx, presenting as oval ulcers with a pseudomembrane and erythematous margins [3]. Aseptic abscess syndrome clinically presents with fever, weight loss, and abdominal pain due to deep abscesses in various organs such as spleen (71,8%), lymph nodes (50,7%), liver (28.1%), lung (22.5%), and skin (29.5%) [6]. Skin lesions in this syndrome resemble those of pyoderma gangrenosum or Sweet’s syndrome—60% of the patients with this syndrome present with IBD symptoms [6].

Skin lesions associated with IBD include erythema nodosum. Erythema nodosum occurs in 3%–10% of patients with chronic ulcerative colitis and 4%–15% of patients with Crohn’s disease (most often with colonic involvement). It is more common in women and typically appears during acute IBD episodes, resulting from a delayed‐type hypersensitivity reaction [5]. Clinically, erythema nodosum presents as red, hot, painful nodules measuring 1–5 cm that do not suppurate or ulcerate. As they resolve, the lesions turn slightly yellow. They are commonly found on the anterior shins, knees, ankles, arms, or torso. Psoriasis is another skin condition frequently associated with IBD, affecting 7%–11% of the patients. This association is due to a shared pathogenesis involving Th1 lymphocytes and proinflammatory cytokines such as TNF‐α, IFN‐γ, and IL‐12. Psoriasis is more common in Crohn’s disease than in chronic ulcerative colitis. Psoriasis typically precedes IBD, and its progression is often unrelated to the inflammatory activity of IBD. Epidermolysis bullosa acquisita (EBA) is a rare skin condition associated with IBD, characterized by subepidermal blisters that rupture and scar. It commonly occurs in trauma‐prone areas such as the hands, knees, and feet. The pathogenesis involves autoantibodies (IgG) against Type VII collagen, which anchors the epidermis to the dermis, leading to inflammation and tissue damage [5]. Other less common skin manifestations include small vessel vasculitis or polyarteritis nodosa [5].

Secondary skin lesions related to IBD treatment can include injection site reactions, severe allergic reactions such as angioedema, anaphylaxis, Stevens–Johnson syndrome, or skin infections due to bacterial superinfection (cellulitis and abscess), viral infection (herpes and papillomavirus), or fungal infection [3, 4]. Nonmelanoma skin cancer may also occur. Mucocutaneous lesions due to nutritional deficiencies associated with IBD can include aphthous ulcers, hair loss, and nail changes.

In our patient, despite the absence of typical IBD symptoms such as diarrhea, bloody stools, or abdominal pain, the presence of pyoderma gangrenosum prompted further investigation. Literature indicates that 50% of pyoderma gangrenosum cases are associated with IBD, leading us to perform a colonoscopy. This confirmed the diagnosis of chronic ulcerative colitis presenting with pyoderma gangrenosum. The prognosis of IBD in this patient depends on several factors indicative of severe disease, such as early onset, extraintestinal manifestations, and extensive colonic involvement despite mild inflammatory activity. Considering these factors, we treated the patient with the TNF‐α inhibitor infliximab. Treatment with infliximab successfully controlled colonic inflammation, led to complete healing of the skin lesions, restored the patient’s preillness body weight, and enabled the patient to fully resume normal daily activities. This case highlights the importance of recognizing extraintestinal manifestations in IBD patients and considering IBD in patients with unexplained lesions in other organs, such as the skin, joints, or eyes, especially when they do not respond to standard treatments.

## 4. Conclusion

The case of a patient with pyoderma gangrenosum diagnosed with chronic ulcerative colitis demonstrates that extraintestinal manifestations, particularly skin and mucosal lesions, are not uncommon in patients with IBD. These manifestations can precede gastrointestinal symptoms. To ensure early and timely diagnosis and select appropriate treatment regimens, it is essential to recognize that IBD is one of the underlying causes of these symptoms. This approach helps control both the intestinal and extraintestinal manifestations of IBD.

## Consent

Consent to publish this case report was obtained.

## Conflicts of Interest

The authors declare no conflicts of interest.

## Author Contributions

Long Cong Nguyen, Nam Hoai Nguyen, Tan Thi Tran, and Trang Thu Khuc designed the study and collected and analyzed the data. Hieu Van Nguyen helped with data collection and analysis. Yen Thi Lo and Ha Thi‐Ngoc Doan contributed to the study design and helped interpret the results. All authors contributed to writing the manuscript.

## Funding

No funding was received for this research.

## Data Availability

The datasets generated and/or analyzed during the current study are available from the corresponding author upon reasonable request.
